# Supplementary material for: Financial cost of assisted reproductive technology for patients in high-income countries: A systematic review protocol
Source: PLoS One. 2025 Feb 13;20(2):e0318780. doi: 10.1371/journal.pone.0318780 (PMC11825044; doi:10.1371/journal.pone.0318780)
Supplement: S1 File — (DOCX) [file pone.0318780.s002.docx]

**SI File: Provisional search strings for various databases**

| **Database** | **Search Terms** |
| --- | --- |
| **PubMed** | (((("health expenditures"[MeSH Major Topic] OR "fees and charges"[MeSH Major Topic] OR ("cost*"[Title/Abstract] OR "expense*"[Title/Abstract] OR "out-of-pocket"[Title/Abstract])) AND ("reproductive techniques, assisted"[MeSH Major Topic] OR "fertilization in vitro"[MeSH Major Topic] OR "infertility/therapy"[MeSH Major Topic] OR ("infertility treatment"[Title/Abstract:~2] OR "fertility treatment"[Title/Abstract:~2] OR "assisted reproducti*"[Title/Abstract] OR "IVF"[Title/Abstract] OR "in vitro fertili*"[Title/Abstract] OR "intra-uterine insemination"[Title/Abstract] OR "embryo transfer"[Title/Abstract]))) NOT ("developing countries"[MeSH Terms] OR "Africa South of the Sahara"[MeSH Terms])) NOT ("animals"[MeSH Terms] NOT "humans"[MeSH Terms])) AND (2001:2023[pdat]) Filters: Humans |
| **EMBASE** | (((exp *"health care cost"/ or cost of reproduction/ or exp "drug cost"/ or exp fee/ or "hospital costs".sh. or "health expenditure*or out-of-pocket".ti,ab,kf.) and assisted reproductive techn*.sh.) or exp *in vitro fertilization/ or exp "infertility therapy"/ or ((infertility or fertility) adj2 treatment*).mp. or "assisted reproducti*".mp. or "IVF".mp. or "in vitro fertili*".mp. or "intra-uterine insemination".mp. or "embryo transfer".ti,ab,kf.) and ("developed countr*" not "developing countr*").sh.  limit 9 to (human and "remove preprint records" and yr="2001 - 2023") |
| **CINAHL** | ((MM "Health Care Costs+") OR (MM "Fees and Charges+") OR TI ( "cost*" or "expense*" or "out-of-pocket" ) OR AB ( "cost*" or "expense*" or "out-of-pocket" ) ) AND ((MM "Reproduction Techniques+") OR (MM "Fertilization in Vitro") OR (MM "Infertility+/TH") OR TI (“infertility N2 treatment” or “fertility N2 treatment” or OR AB infertility N2 treatment or "assisted reproducti*" or "IVF" or "in vitro fertili*" or "intra-uterine insemination" or "embryo transfer") OR AB (“infertility N2 treatment” or “fertility N2 treatment” or OR AB infertility N2 treatment or "assisted reproducti*" or "IVF" or "in vitro fertili*" or "intra-uterine insemination" or "embryo transfer")) NOT (MM "Low and Middle Income Countries" OR "Developing Countries") NOT (MM "animals" AND MM "humans")  **Limiters** - Publication Date: 20010101-20231231; Human; |
| **Web of Science** | ((TS=(“health expenditure*” OR "fees and charges") OR TI=(cost* or expense* or "out-of-pocket”) OR AB=(cost* or expense* or "out-of-pocket”)) AND TS=(“Assisted reproductive techn*” or “in vitro fertilization in” or “Infertility therapy”) OR TI=((infertility OR fertility) NEAR/2 treatment* OR "assisted reproducti*" OR "IVF" OR "in vitro fertili*" OR "intra-uterine insemination" OR “frozen embryo transfer" ) OR AB=((infertility OR fertility) NEAR/2 treatment* OR "assisted reproducti*" OR "IVF" OR "in vitro fertili*" OR "intra-uterine insemination" OR “frozen embryo transfer" )) AND TS=(“high income countr*” or “developed countr*”) NOT TS=("Low and Middle Income Countries" OR "Developing Countries") NOT TS=(animals)  ***Timespan:*** 2001-01-01 to 2023-12-31 (Index Date) |
